# TRIM6 Reduces Ferroptosis and Chemosensitivity by Targeting SLC1A5 in Lung Cancer

**DOI:** 10.1155/2023/9808100

**Published:** 2023-01-09

**Authors:** Ying Zhang, Ping Dong, Nian Liu, Jun-Yuan Yang, Hui-Min Wang, Qing Geng

**Affiliations:** ^1^Department of Vascular Surgery, Renmin Hospital of Wuhan University, Wuhan, 430060 Hubei, China; ^2^Department of Thoracic Surgery, Renmin Hospital of Wuhan University, Wuhan, 430060 Hubei, China; ^3^Department of Neonatology, Renmin Hospital of Wuhan University, Wuhan, 430060 Hubei, China; ^4^Department of Gynecologic Oncology, Zhongnan Hospital of Wuhan University, Wuhan, 430071 Hubei, China; ^5^Department of Fever Clinic, Renmin Hospital of Wuhan University, Wuhan, 430060 Hubei, China

## Abstract

**Objective:**

Ferroptosis, a newly identified form of cell death, plays critical roles in the development and chemoresistance of lung cancer. Tripartite motif 6 (TRIM6) acts as an E3-ubiquitin ligase and can promote the progression of human colorectal cancer. The present study is aimed at investigating its role and potential mechanisms in lung cancer.

**Methods:**

Lentiviral vectors were used to overexpress or knock down TRIM6 in human lung cancer cells. Cell survival, colony formation, lipid peroxidation, intracellular iron levels, and other ferroptotic markers were examined. The role of TRIM6 on ferroptosis and chemosensitivity was further tested in mouse tumor xenograft models.

**Results:**

TRIM6 was highly expressed in human lung cancer tissues and cells, and its expression in the lung cancer cells was further increased by ferroptotic stimulation. TRIM6 overexpression inhibited, while TRIM6 silence promoted erastin- and RSL3-induced glutaminolysis and ferroptosis in the lung cancer cells. Mechanistically, TRIM6 directly interacted with solute carrier family 1 member 5 to promote its ubiquitination and degradation, thereby inhibiting glutamine import, glutaminolysis, lipid peroxidation, and ferroptotic cell death. Moreover, we observed that TRIM6 overexpression reduced the chemotherapeutic effects of cisplatin and paclitaxel. In contrast, TRIM6 silence sensitized human lung cancer cells to cisplatin and paclitaxel in vivo and in vitro.

**Conclusion:**

Our findings for the first time define TRIM6 as a negative regulator of ferroptosis in the lung cancer cells, and TRIM6 overexpression enhances the resistance of human lung cancer cells to chemotherapeutic drugs. Overall, targeting TRIM6 may help to establish novel strategies to treat lung cancer.

## 1. Introduction

Lung cancer is the leading cause of cancer mortality worldwide, and most patients are diagnosed at the advanced stages, with very poor prognosis [1–5]. Cell death plays an important role in regulating tumor growth, progression, and chemotherapeutic response. Ferroptosis is a newly discovered nonapoptotic death mode that involves the accumulation of lipid reactive oxygen species (ROS) and subsequent depletion of plasma membrane polyunsaturated fatty acids [6–10]. Glutathione (GSH) and the associated glutathione peroxidase 4 (GPX4) are intracellular antioxidant defenses to scavenge the toxic lipid ROS [11]. In contrast, iron donates electrons to oxygen to accelerate lipid ROS formation and ferroptosis [12]. Accordingly, lipophilic or membrane impermeable iron chelators notably prevent lethal lipid peroxidation and ferroptosis [11, 13]. L-Glutamine (Gln) is a major nitrogen source for the synthesis of amino acids, nucleotides, and lipids and also provides carbon source for the tricarboxylic acid (TCA) cycle and cellular energetics, which is required for the growth of cancer cells [14]. Yet, recent findings have found that glutaminolysis promotes productions of oxidizable lipids via the TCA cycle and eventually facilitates ferroptosis [15, 16]. Gln is imported inside the cells by solute carrier family 1 member 5 (SLC1A5) and SLC38A1, converted into glutamate (Glu) by glutaminases (GLS), and then metabolized into alpha-ketoglutarate (*α*-KG) by either glutamate dehydrogenase- (GLUD1-) mediated glutamate deamination or glutamic-oxaloacetic transaminase 1 (GOT1-) mediated transamination [15]. And supplementing *α*-KG can fuel both energetic and anabolic pathways, mimicking Gln-mediated ferroptotic induction. Therefore, targeting ferroptosis may develop novel therapeutic approaches to treat lung cancer.

Ubiquitination acts as a pivotal posttranslational modification for various proteins. During ubiquitination, polyubiquitin (Ub) chains are attached to the targeted proteins by E1 Ub-activating enzymes, E2 Ub-conjugating enzymes, and E3 Ub-ligases, which then mediate the proteasomal degradation of these proteins [17–20]. Tripartite motif (TRIM) proteins are a family of E3 Ub-ligases and implicated in carcinogenesis and chemoresistance of diverse cancers [21–23]. TRIM6, a member of TRIM proteins, plays critical roles in regulating interferon signaling and antiviral responses [24, 25]. Results from Zeng et al. demonstrated that TRIM6 aggravated cardiomyocyte apoptosis and myocardial ischemia/reperfusion injury [26]. TRIM6 could also interact with protooncogenic Myc to maintain the pluripotency of mouse embryonic stem cells [27]. Moreover, Zheng et al. recently observed that TRIM6 was upregulated in human colorectal cancer (CRC) samples and that TRIM6 overexpression promoted proliferation and chemoresistance of CRC cells [28]. These findings identify TRIM6 as a promising therapeutic target of lung cancer.

## 2. Materials and Methods

### 2.1. Antibodies and Chemicals

Anti-TRIM6 (#11953-1-AP) and anti-glyceraldehyde-3-phosphate dehydrogenase (GAPDH, #10494-1-AP) were purchased from Proteintech (Chicago, IL, USA). Anti-GPX4 (#ab125066), anti-SLC7A11 (xCT, #ab37185), anti-SLC3A2 (CD98, #ab108300), anti-glutathione synthetase (GSS, #ab124811), anti-transferrin (Tf, #ab109503), anti-Tf receptor (TfR, #ab84036), anti-nuclear factor E2-related factor 2 (NRF2, #ab137550), anti-SLC1A5 (#ab237704), and anti-SLC38A1 (#ab60145) were purchased from Abcam (Cambridge, UK), while anti-ferroportin (FPN, #NBP1-21502) was obtained from Novus Biologicals (Littleton, Colorado, USA). Erastin (#S7242), RSL3 (#S8155), ferrostain-1 (Fer-1, #S7243), and liproxstatin-1 (Lip-1, #S7699) were obtained from Selleck Chemicals (Houston, TX, USA). 2′,7′-dichlorofluorescin diacetate (DCFH-DA, #D6883), superoxide anion assay kit (#CS1000), GSH assay kits (#CS0206), *α*-KG (#349631), L-*γ*-glutamyl transpeptidase substrate (SLC1A5 inhibitor; GPNA, #G1135), compound 968 (GLS inhibitor; 968, #352010), bis-2-(5-phenylacetamido-1,3,4-thiadiazol-2-yl) ethyl sulfide (GLS1 inhibitor; BPTES, #SML0601), amino oxyacetate (pan-transaminase inhibitor; AOA, #C13408), cycloheximide (protein synthesis inhibitor; CHX, #01810), MG132 (proteasome inhibitor, #M7449), cisplatin (DDP, #P4394), and paclitaxel (PTX, #1491332) were purchased from Sigma-Aldrich (St. Louis, MO, USA). BODIPY™ 581/591 C11 (BODIPY, #D3861) and tetramethylrhodamine ethyl ester (TMRE, #T669) were obtained from Invitrogen (Carlsbad, CA, USA). Malondialdehyde (MDA) assay kits (#ab118970) were purchased from Abcam (Cambridge, UK), while CellTiter 96® AQ_ueous_ One Solution Cell Proliferation Assay kit (MTS assay, #G3582) was obtained from Promega (Madison, WI, USA). Lentivirus carrying the short hairpin RNA sequences against human TRIM6 (*TRIM6*-KD #1 and *TRIM6*-KD #2), human SLC1A5 (*SLC1A5*-KD), or the control sequence (*CTRL*-KD) were generated by Gene Pharma Corporation (Shanghai, China). For TRIM6 overexpression, human TRIM6 cDNA (*TRIM6*-OE), human SLC1A5 cDNA (*SLC1A5*-OE), or a negative control (*CTRL*-OE) sequence was cloned into the lentiviral vectors by Gene Pharma Corporation (Shanghai, China).

### 2.2. Cell Culture

Human lung cancer cell lines A549, H358, H460, H1299, PC9, and SPC-A-1 and normal human lung epithelial cell BEAS-2B were purchased from American Type Culture Collection and cultured in DMEM medium supplemented with 10% fetal bovine serum (FBS) and 1% antibiotics at 37°C under the humidified atmosphere [29–31]. The cells were preinfected for 12 h with lentiviral vectors carrying two different interfering sequences against TRIM6 at a multiplicity of infection (MOI) of 50 to silence endogenous TRIM6 or with *TRIM6*-OE virus (MOI = 20) to overexpress TRIM6. And then, the cells were maintained in fresh medium containing 10% FBS for an additional 24 h before further treatment. To induce ferroptosis, the cells were incubated with erastin (5 *μ*mol/L) or RSL3 (2 *μ*mol/L) for 24 h after TRIM6 genetic manipulation except special annotation [32]. For ferroptosis suppression, Fer-1 (1 *μ*mol/L) or Lip-1 (0.2 *μ*mol/L) was added at 8 h before erastin or RSL3 treatment [33]. In addition, the cells were treated with GPNA (5 mmol/L), 968 (20 *μ*mol/L), BPTES (10 *μ*mol/L), or AOA (0.5 mmol/L) at 8 h before erastin or RSL3 stimulation to inhibit Gln uptake or metabolism in the presence or absence of *α*-KG (4 mmol/L) [15, 16]. For SLC1A5 overexpression or silence, the cells were preinfected with *SLC1A5*-OE (MOI = 20) or *SLC1A5*-KD (MOI = 50) for 12 h before TRIM6 genetic manipulation. In a separated study, the cells were infected with *TRIM6*-OE (MOI = 20) or *CTRL*-OE for 12 h and then cultured in normal medium for an addition 24 h, followed by a stimulation with CHX (20 mmol/L) for indicating times [28]. To clarify the role of TRIM6 on chemosensitivity in human lung cancer cells, the cells were treated with DDP (20 *μ*mol/L) or PTX (0.3 *μ*mol/L) for 12 h after TRIM6 genetic manipulation [34].

### 2.3. Cell Survival Assay

Cell survival was determined using the CellTiter 96® AQ_ueous_ One Solution Cell Proliferation Assay kit (MTS assay) [32]. Briefly, the cells (approximately 200 cells in 96-well plates) were incubated with CellTiter 96® AQ_ueous_ One Solution Reagent (20 *μ*L per 100 *μ*L medium) at 37°C for 2 h under the humidified atmosphere, and then, the absorbance was recorded at 490 nm using a 96-well plate reader.

### 2.4. Colony Formation Assay

For colony formation assay, the cells were seeded into the 6-well plates and incubated for 14 days with the colonies stained by 0.1% crystal violet. Next, the colonies were carefully rinsed with tap water and dried at room temperature, and the colonies with a diameter more than 0.05 mm were counted by ImageJ software in a blinded manner [32, 35, 36].

### 2.5. Measurements of Intracellular ROS and Lipid Peroxidation

Intracellular ROS production was measured using the nonfluorescent DCFH-DA reagent that could be converted to the fluorescent DCF by free radicals [37–39]. In brief, the cells were homogenized in the assay buffer and then incubated with DCFH-DA (10 *μ*mol/L) at 37°C for 30 min. The fluorescent intensity was examined using a spectrofluorometer with an excitation/emission wavelength at 488/525 nm. To detect lipid ROS level, the cells were incubated with BODIPY (10 *μ*mol/L) at 37°C for 30 min and the fluorescent intensity was recorded by the simultaneous acquisition of green signals (484/510 nm) and red signals (581/610 nm) using the BD FACSAria cytometer [32]. Intracellular MDA content was assessed using the commercial kit following the manufacturer's instructions, and the absorbance was measured at 532 nm [32, 40].

### 2.6. Evaluations of GSH Level and GPX4 Activity

Intracellular GSH level was evaluated with a commercial kit according to the manufacturer's protocols and assayed colorimetrically at 412 nm. Relative GPX4 activity was determined using the HPTLC method according to previous studies [11, 41]. In brief, the cells were lysed in the reaction buffer, and the supernatants were collected to incubate with 7*α* cholesterol hydroperoxide (100 *μ*mol/L) at 37°C. Next, the peroxides were extracted for HPTLC analysis, and analytes were scanned and quantified using ImageJ software.

### 2.7. Detections of Superoxide Anion Generation and Mitochondrial Membrane Potential (MMP)

Superoxide anion generation was assessed with a superoxide anion assay kit via referring to the standard protocols. Briefly, the cells were incubated with luminol solution (5 *μ*L) and enhancer solution (5 *μ*L) at 37°C for 15 min, and then, the luminescence intensity was immediately measured. MMP was measured by incubating the cells with TMRE (200 nmol/L) at 37°C for 20 min, and the fluorescence intensity of TMRE was determined at 582 nm [42].

### 2.8. Iron Assay

Labile iron pool (LIP) was measured by the calcein-acetoxymethyl ester method [43]. In brief, intracellular LIP was loaded with calcein (2 *μ*mol/L) at 37°C for 30 min, and then, the calcein was removed from iron by deferoxamine (100 *μ*mol/L). The changes of fluorescence intensity with or without deferoxamine incubation at an excitation/emission wavelength of 485/535 nm were quantified as the amount of LIP. Ferrous iron (Fe^2+^) levels were quantified at 593 nm using a commercial kit.

### 2.9. Protein Extraction, Immunoblots (IB), and Immunoprecipitation (IP)

Cells were lysed in the RIPA lysis buffer containing protease/phosphatase inhibitor cocktail at 4°C, and total protein concentrations were determined by the bicinchoninic acid kit [44–46]. Then, equal amounts of proteins were separated by sodium dodecyl sulfate/polyacrylamide gels (SDS-PAGE) and electrotransferred to the polyvinylidene difluoride membranes, followed by an incubation with 5% nonfat dried milk to block nonspecific binding. Next, the membranes were incubated with primary antibodies at 4°C overnight and stained by the secondary antibodies at room temperature for an additional 1 h. After that, protein bands were visualized with an ECL reagent and analyzed using the ImageJ software. For IP assay, cells were lysed in IP lysis buffer, and then, the lysates were incubated with indicating primary antibodies or IgG at 4°C overnight with gentle shaking, followed by the incubation with Protein A/G-agarose beads at room temperature for an additional 2 h. The immunoprecipitated proteins were subsequently washed for 5 times using the lysis buffer and boiled before SDS-PAGE electrophoresis.

### 2.10. RNA Purification and Quantitative Real-Time PCR

Total RNA was extracted using TRIzol reagent and then converted to cDNA using oligo (dT) primers. Quantitative real-time PCR was performed using QuantiNova SYBR Green PCR Kit (Qiagen; Hamburg, Germany) and normalized to *GAPDH* gene expression [47, 48].

### 2.11. Gln Uptake Assay

Gln uptake assay was performed using the [^3^H]-L-Gln according to a previous study [15]. In brief, the cells were incubated with [^3^H]-L-Gln (200 nmol/L) in Gln-free medium at 37°C for 15 min, which were then harvested for Gln measurements using a liquid scintillation counter.

### 2.12. Ubiquitination Assay In Vivo and In Vitro

For the in vivo ubiquitination assay, HEK293T cells were transfected with indicating plasmids for 48 h, and then, the cells were harvested in lysis buffer. Next, the samples were incubated with HA beads at 4°C for 2 h and then subjected to IP assay. For the in vitro ubiquitination assay, purified HA-SLC1A5 proteins were incubated with E1, E2 enzymes and human recombinant Ub with or without Flag-TRIM6 proteins in ubiquitination reaction buffer (Boston Biochem) at 30°C for 90 min, and then, the samples were prepared for IP assay [49, 50].

### 2.13. Mouse Xenograft Tumor Model

All animal experiments were approved by the Animal Ethics Committee of Renmin Hospital of Wuhan University and also complied with the *Animal Research: Reporting of In Vivo Experiments* (ARRIVE) guidelines. 5 × 10^6^ TRIM6-manipulated H460 or PC9 cells were subcutaneously inoculated into the right flank of athymic BALB/c nude mice (4-5 weeks old), and the tumor parameters were calculated 4 weeks after cell inoculation [15]. To validate the role of TRIM6 on chemosensitivity, tumor-bearing mice received intraperitoneal injections of DDP (5 mg/kg) or PTX (15 mg/kg) for 3 times every other day at the last week before study termination [51].

### 2.14. Human Tissue Samples

Lung adenocarcinoma (ADC), squamous cell cancer (SCC), and corresponding adjacent normal tissues (ANT) were obtained from the patients without neoadjuvant or adjuvant therapies after written informed consent signed. ANT was obtained from the same patients and was at least 3 cm away from the tumor tissue. This study was approved by the Institutional Review Board of Renmin Hospital of Wuhan University and conformed to the principles outlined in the *Declaration of Helsinki*.

### 2.15. Statistical Analysis

All data are reported as the mean ± SD, and *P* < 0.05 was considered statistically significant. Differences between two groups were compared using Student's two-tailed *t*-test, while one-way ANOVA followed by the Tukey post hoc test was applied for comparison of multiple groups. All statistical analyses were performed using SPSS 19.0 software in a blinded manner.

## 3. Results

### 3.1. TRIM6 Expression in the Lung Cancer Samples Is Increased upon Ferroptotic Stimulation

**Figure 1:**
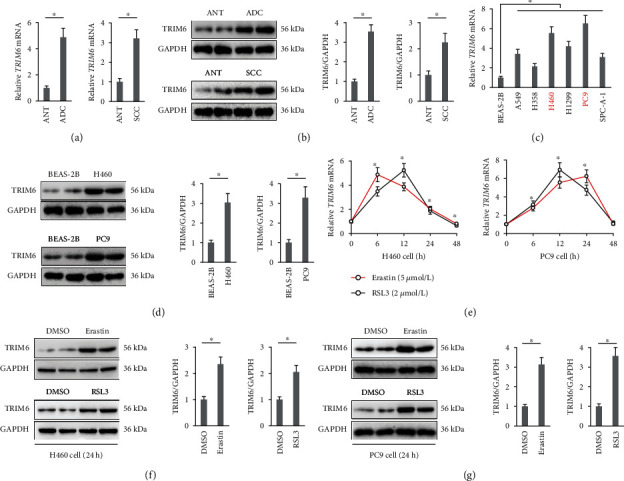
TRIM6 expression in lung cancer samples is increased upon ferroptotic stimulation. (a) Relative *TRIM6* mRNA levels in ADC, SCC, and corresponding ANT (*n* = 10). (b) Protein levels of TRIM6 in ADC, SCC, and corresponding ANT (*n* = 6). (c) Relative *TRIM6* mRNA levels in human lung cancer cell lines and normal epithelial cell (*n* = 6). (d) Protein levels of TRIM6 in H460, PC9, and BEAS-2B cells (*n* = 6). (e) Relative *TRIM6* mRNA levels in erastin- or RSL3-treated human lung cancer cells (*n* = 6). (f, g) Protein levels of TRIM6 in erastin- or RSL3-treated human lung cancer cells (*n* = 6). All data are reported as the mean ± SD, ^∗^*P* < 0.05 versus corresponding groups.

We first compared TRIM6 expression in human lung cancer tissues and corresponding ANT. As shown in Figures 1(a) and 1(b), human lung ADC and SCC tissues exhibited higher TRIM6 expression. Besides, *TRIM6* mRNA levels were also increased in serials of the lung cancer cell lines (A549, H358, H460, H1299, PC9, and SPC-A-1) in comparison with the normal human lung epithelial cell BEAS-2B (Figure 1(c)). Besides, we found that *TRIM6* mRNA expressions in H460 and PC9 cells were higher than those in other cancer cell lines; therefore, we selected these two cell lines in our further study (Figure 1(c)). Consistent with the mRNA levels, increased TRIM6 protein expressions were also detected in H460 and PC9 cells compared with BEAS-2B cell (Figure 1(d)). Next, we explored whether TRIM6 expression in the lung cancer cells was altered upon ferroptotic stimulation. As shown in Figure 1(e), *TRIM6* mRNA levels in H460 and PC9 cells were increased in the initial phase after erastin or RSL3 treatment, but fell and even decreased at the later stages. Therefore, all cells were incubated with erastin or RSL3 for 24 h except special annotation in our further experiments. At this time, both of the two cell lines had increased TRIM6 expression and also received sufficient intensities of ferroptotic stimulation (Figure 1(e)). Results from IB further confirmed that TRIM6 expression in the lung cancer cells was increased upon ferroptotic stimulation (Figures 1(f) and 1(g)). Collectively, these data demonstrate a potential involvement of TRIM6 in ferroptosis of the lung cancer cells.

### 3.2. TRIM6 Overexpression Inhibits Erastin- and RSL3-Induced Ferroptosis in the Lung Cancer Cells

**Figure 2:**
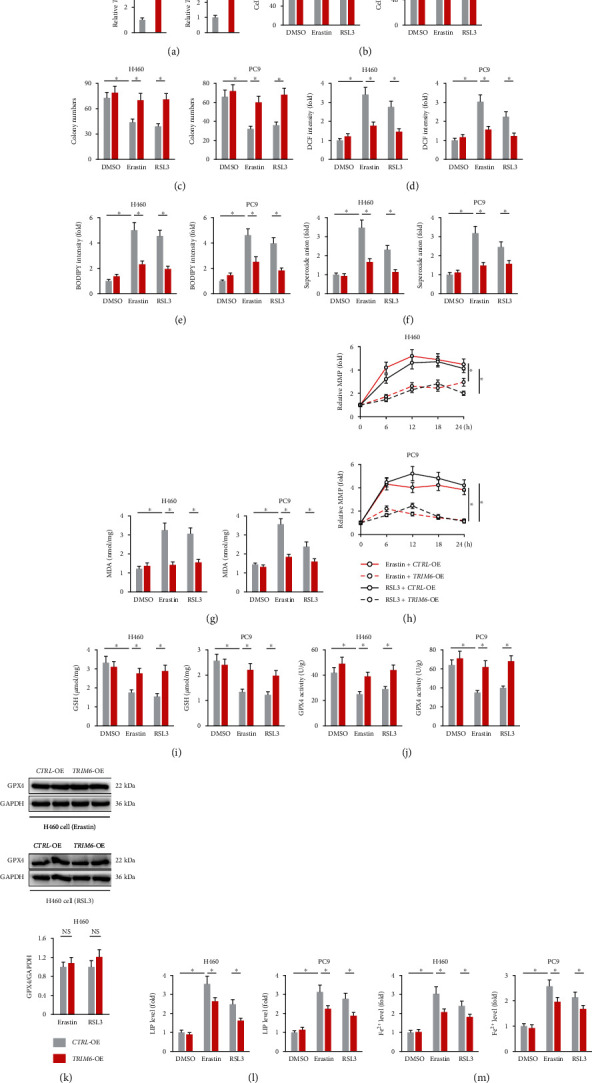
TRIM6 overexpression inhibits erastin- and RSL3-induced ferroptosis in the lung cancer cells. (a) Relative *TRIM6* mRNA levels in H460 and PC9 cells with or without *TRIM6*-OE infection (*n* = 6). (b) Cell survival status in the presence or absence of erastin/RSL3 stimulation after TRIM6 overexpression (*n* = 6). (c) Colony formation in the cells with or without TRIM6 overexpression upon ferroptotic stimulation (*n* = 6). (d, e) Intracellular ROS and lipid peroxidation levels (*n* = 6). (f, g) The levels of intracellular superoxide anion and MDA formation (*n* = 6). (h) Relative MMP levels in indicating times after erastin/RSL3 stimulation (*n* = 5). (i, j) The levels of intracellular GSH and GPX4 activities (*n* = 6). (k) Protein levels of GPX4 in erastin- or RSL3-treated lung cancer cells after TRIM6 overexpression (*n* = 6). (l, m) Relative LIP and Fe^2+^ levels (*n* = 6). All data are reported as the mean ± SD, ^∗^*P* < 0.05 versus corresponding groups.

We then overexpressed TRIM6 in H460 and PC9 cells using lentiviral vectors, and the efficiency was confirmed in Figure 2(a). Interestingly, TRIM6 overexpression significantly enhanced the survival and colony formation of the lung cancer cells upon ferroptotic stimulation (Figures 2(b) and 2(c)). Lipid peroxidation is an important feature of ferroptosis [13]. As expected, erastin and RSL3 treatment provoked significant increases of cellular and lipid ROS production, which were inhibited in TRIM6-overexpressed cells (Figures 2(d) and 2(e)). The levels of intracellular superoxide anion and MDA generation were also decreased by TRIM6 overexpression (Figures 2(f) and 2(g)). Consistent with previous studies, the lung cancer cells with erastin and RSL3 treatment exhibited higher MMP levels that were inhibited by TRIM6 overexpression (Figure 2(h)) [52]. GSH and GPX4 are essential for reducing lipid hydroperoxides to lipid alcohols, thereby preventing lipid peroxidation and ferroptotic cell death [11]. As shown in Figures 2(i) and 2(j), the cells with erastin or RSL3 stimulation exhibited lower levels of GSH and GPX4 activities, which were preserved by TRIM6 overexpression. However, TRIM6 overexpression did not affect GPX4 protein abundances upon ferroptotic stimulation (Figure 2(k)). Iron, especially LIP and Fe^2+^, is essential for the execution of ferroptosis, and we thus evaluated the effect of TRIM6 on intracellular LIP and Fe^2+^ levels [13, 43]. We observed that TRIM6 overexpression slightly but significantly reduced iron accumulation following the treatment with erastin or RSL3 (Figures 2(l) and 2(m)). These findings suggest that TRIM6 overexpression inhibits erastin- and RSL3-induced ferroptosis in the lung cancer cells.

### 3.3. TRIM6 Silence Promotes Erastin- and RSL3-Induced Ferroptosis in the Lung Cancer Cells

**Figure 3:**
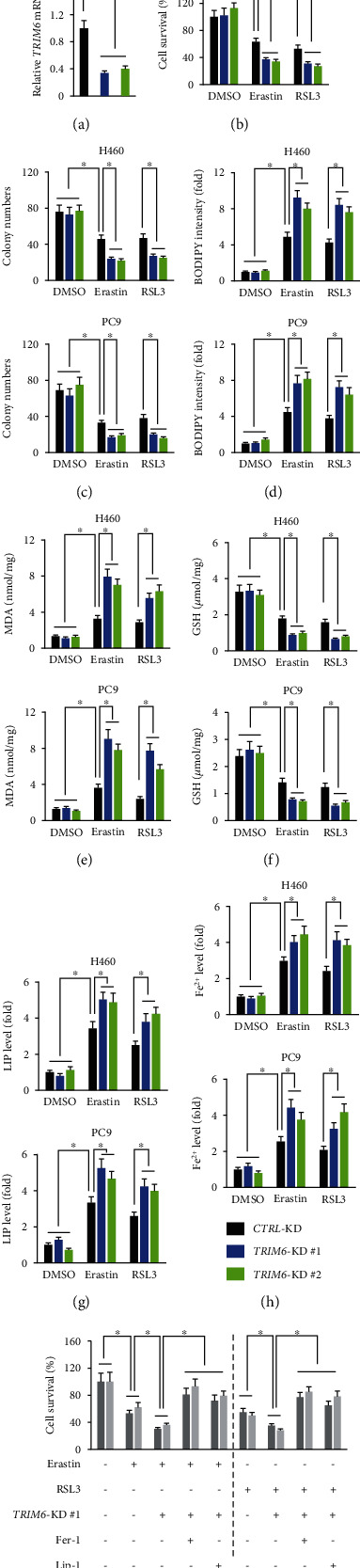
TRIM6 silence promotes erastin- and RSL3-induced ferroptosis in the lung cancer cells. (a) Relative *TRIM6* mRNA levels in H460 and PC9 cells with or without *TRIM6*-KD infection (*n* = 6). (b) Cell survival status in the presence or absence of erastin/RSL3 stimulation after TRIM6 knockdown (*n* = 6). (c) Colony formation in the cells with or without TRIM6 silence upon ferroptotic stimulation (*n* = 6). (d, e) Intracellular lipid ROS levels and MDA formation (*n* = 6). (f) Intracellular GSH levels (*n* = 6). (g, h) Relative LIP and Fe^2+^ levels (*n* = 6). (i) Cell survival status (*n* = 6). All data are reported as the mean ± SD, ^∗^*P* < 0.05 versus corresponding groups.

Next, we used two lentiviral vectors to knock down endogenous TRIM6 expression, and the efficiency was confirmed in Figure 3(a). As expected, TRIM6 silence further decreased the survival and colony formation of the lung cancer cells upon erastin and RSL3 treatment (Figures 3(b) and 3(c)). Lipid ROS level and MDA generation were also augmented in TRIM6-deficient cells (Figures 3(d) and 3(e)). GSH depletions in H460 and PC9 cells by erastin or RSL3 incubation were more obvious after TRIM6 knockdown (Figure 3(f)). Intracellular LIP and Fe^2+^ levels were increased in the lung cancer cells by ferroptotic stimulation, which were further enhanced in those with TRIM6 silence (Figures 3(g) and 3(h)). However, TRIM6 knockdown-associated cell death could be remarkably suppressed by ferroptosis inhibitors, Fer-1 and Lip-1, indicating an involvement of ferroptosis (Figure 3(h)). These data imply that TRIM6 silence promotes erastin- and RSL3-induced ferroptosis in the lung cancer cells.

### 3.4. TRIM6 Modulates Ferroptosis via Affecting SLC1A5-Mediated Glutaminolysis

**Figure 4:**
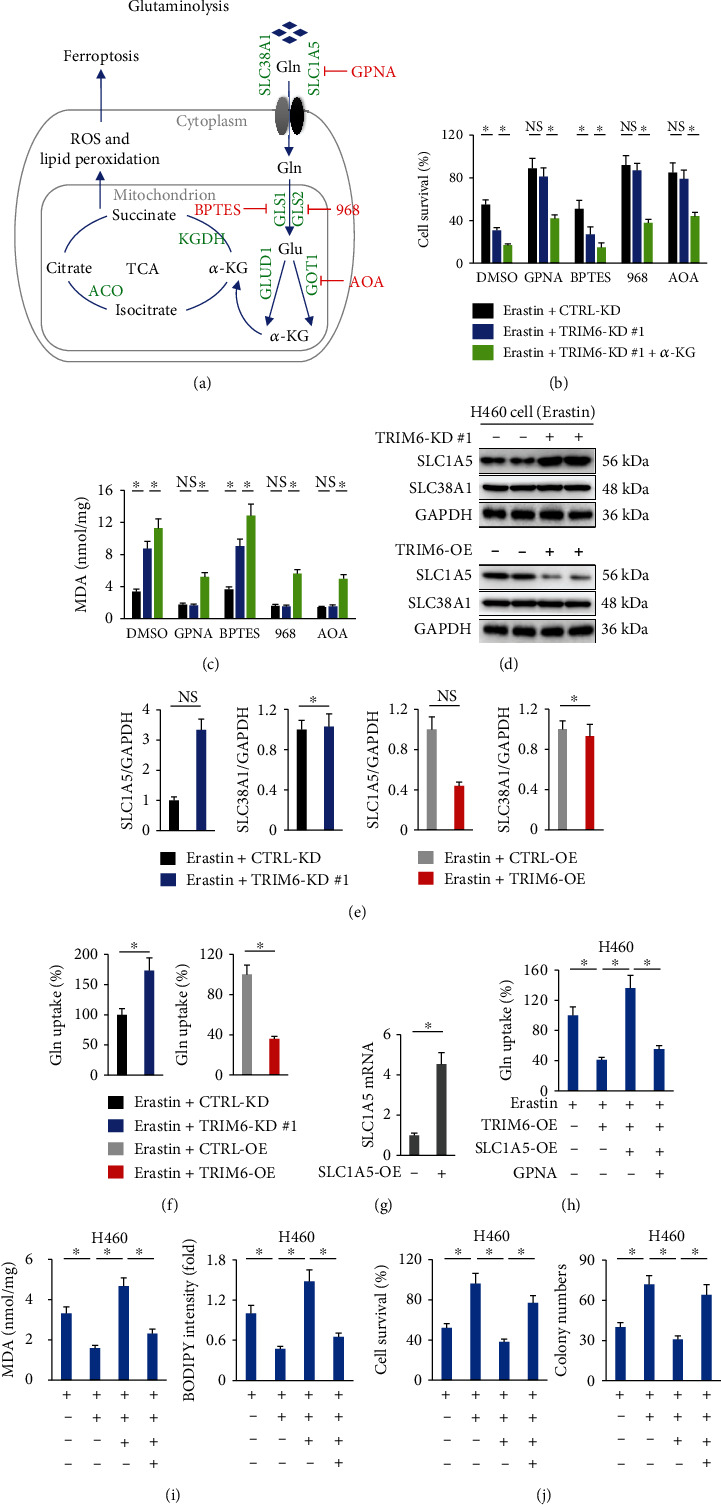
TRIM6 modulates ferroptosis via affecting SLC1A5-mediated glutaminolysis. (a) Schematic overview of the glutaminolysis pathway and TCA cycle in ferroptosis. Gln is imported inside the cells by SLC1A5/SLC38A1 and then converted to Glu by GLS in mitochondria. The GOT1 and GLUD1 ultimately converts Glu to *α*-KG, which contributes to ROS accumulation via the TCA cycle. The small molecule inhibitors are indicated in red: L-Gln transporter inhibitor, GPNA; GLS1 inhibitor, BPTES; GLS inhibitor, 968; pan-transaminase inhibitor, AOA. (b, c) Cell survival and MDA formation in erastin-treated H460 cells (*n* = 6). (d, e) Protein levels of SLC1A5 and SLC38A1 in erastin-treated H460 cells with TRIM6 knockdown or overexpression (*n* = 6). (f) Relative Gln uptake in erastin-treated H460 cells (*n* = 8). (g) Relative *SLC1A5* mRNA levels in H460 cells with or without *SLC1A5*-OE infection (*n* = 6). (h) Relative Gln uptake in erastin-treated H460 cells (*n* = 8). (i) Intracellular lipid ROS levels and MDA formation in erastin-treated H460 cells (*n* = 6). (j) Cell survival status and colony formation in erastin-treated H460 cells (*n* = 6). All data are reported as the mean ± SD, ^∗^*P* < 0.05 versus corresponding groups. NS indicates no significance.

We then examined the possible molecular basis underlying TRIM6-mediated ferroptotic actions. Unexpectedly, TRIM6 silence did not affect the molecules essential for Glu uptake, GSH synthesis, and iron transport (Figure S1A). In view of the unchangeable GPX4 proteins and slight alterations of the iron accumulation, we speculated that GSH/GPX4-mediated antioxidant defenses and iron overload might not be the primary mechanisms for TRIM6-mediated ferroptotic actions. NRF2 is a major redox-dependent transcription factor and negatively regulates ferroptosis [53]. However, TRIM6 knockdown also unaffected NRF2 expression and its transcription activity, as confirmed by expressions of the downstream heme oxygenase 1 (*HMOX1*), NAD(P)H quinone dehydrogenase 1 (*NQO1*), and glutamate-cysteine ligase modifier subunit (*GCLM*) (Figures S1A and S1B). Gln provides nutrition for the growth of cancer cells; however, recent studies have reported that glutaminolysis is linked to ferroptosis of the cancer cells via inducing the accumulation of lipid ROS accumulation [14–16]. To investigate whether TRIM6 regulated ferroptosis via affecting glutaminolysis, TRIM6-deficient cells were treated with different pharmacological inhibitors of Gln metabolism upon erastin stimulation (Figure 4(a)). As shown in Figures 4(b) and 4(c), cell death and MDA formation in TRIM6-deficient H460 cells were markedly suppressed by inhibitors of Gln metabolism, except BPTES, a specific GLS1 inhibitor. In contrast, supplementation of *α*-KG, the final product of glutaminolysis, reinduced ferroptosis of erastin-treated lung cancer cells in the presence of GPNA, 968, and AOA (Figures 4(b) and 4(c)). SLC1A5 and SLC38A1 are two critical Gln importers and play critical roles in regulating ferroptosis and lung cancer [15]. We found that TRIM6 silence increased, while TRIM6 overexpression decreased SLC1A5 protein levels in erastin-treated H460 cells, with no impact on SLC38A1 expressions (Figures 4(d) and 4(e)). Accordingly, Gln uptake was enhanced in the lung cancer cells with TRIM6 silence, but inhibited in those with TRIM6 overexpression (Figure 4(f)). To further confirm the involvement of SLC1A5, H460 cells were preinfected with *SLC1A5*-OE lentivirus, and the efficiency was confirmed in Figure 4(g). As shown in Figure 4(h), Gln uptake in erastin-treated H460 cells was decreased by TRIM6 overexpression, yet restored by SLC1A5 overexpression, which was inhibited by GPNA incubation. The decreases of MDA and lipid ROS generation in TRIM6-overexpressed cells with erastin stimulation were increased after the overexpression of SLC1A5, which were then suppressed by GPNA treatment (Figure 4(i)). Accordingly, TRIM6 overexpression-mediated restorations of cell survival and colony formation were prevented in SLC1A5-overexpressed H460 cells, but not in those treated with GPNA (Figure 4(j)). In contrast, the increased Gln uptake in TRIM6-deficient cells was significantly inhibited by *SLC1A5*-KD infection (Figures S2A and S2B). Correspondingly, TRIM6 silence-elicited ferroptosis was attenuated in SLC1A5-deficient H460 cells (Figures S2C and S2D). These data indicate that TRIM6 modulates ferroptosis via affecting SLC1A5-mediated glutaminolysis.

### 3.5. TRIM6 Directly Interacts with SLC1A5 to Promote Its Degradation

**Figure 5:**
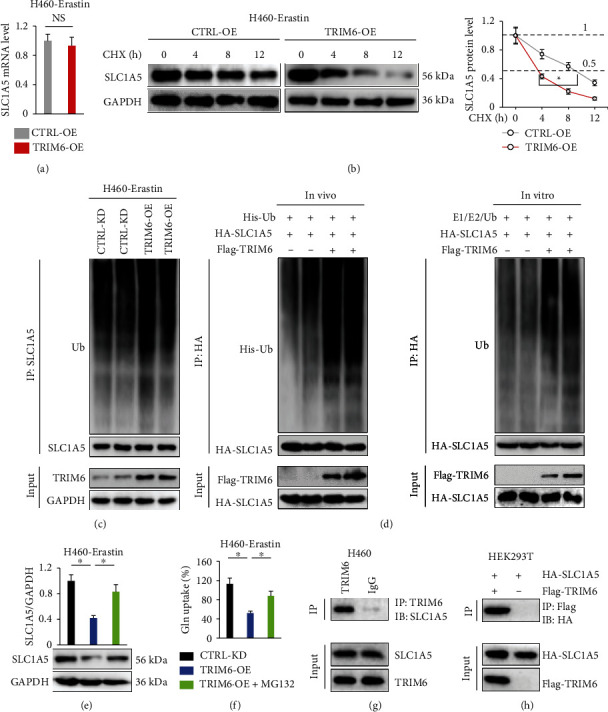
TRIM6 directly interacts with SLC1A5 to promote its degradation. (a) Relative *SLC1A5* mRNA levels in erastin-treated H460 cells with or without *TRIM6*-OE infection (*n* = 6). (b) Protein levels of SLC1A5 in erastin-treated H460 cells after CHX incubation (*n* = 6). (c) Ubiquinated levels of SLC1A5 in erastin-treated cells with or without TRIM6 overexpression (*n* = 6). (d) Ubiquitination assay in vivo and in vitro (*n* = 4). (e) Protein levels of SLC1A5 in erastin-treated H460 cells after MG132 incubation (*n* = 6). (f) Relative Gln uptake in erastin-treated H460 cells after MG132 incubation (*n* = 6). (g, h) IP assay for examining the interaction between TRIM6 and SLC1A5 (*n* = 6). All data are reported as the mean ± SD, ^∗^*P* < 0.05 versus corresponding groups. NS indicates no significance.

We also investigated how TRIM6 regulated SLC1A5 in H460 cancer cells. As shown in Figure 5(a), TRIM6 overexpression made no alteration on *SLC1A5* mRNA level. This finding suggested that SLC1A5 protein might be destabilized in TRIM6-overexpressed cells, and we thus assessed the half-life of SLC1A5 by treating TRIM6-manipulated H460 cells with CHX. As shown in Figure 5(b), TRIM6 overexpression significantly shortened the half-life of SLC1A5 protein. TRIM6 functions as an E3 Ub-ligase, while it is unclear whether TRIM6 affects SLC1A5 protein stability via regulating its ubiquitination. Intriguingly, we found that TRIM6 overexpression enhanced SLC1A5 ubiquitination in erastin-treated H460 cells (Figure 5(c)). The catalytic ability of TRIM6 on SLC1A5 ubiquitination was also confirmed in vivo and in vitro (Figure 5(d)). To determine the Ub-dependent proteasomal degradation of SLC1A5, TRIM6-overexpressed H460 cells were incubated with MG132 upon erastin treatment. The data implied that MG132 treatment blocked the reduction of SLC1A5 proteins caused by TRIM6 overexpression (Figure 5(e)). Accordingly, the decreased Gln uptake was also prevented by MG132 incubation (Figure 5(f)). We next explored whether this ubiquinated process depended on the direct interaction between TRIM6 and SLC1A5. The endogenous physical interaction was confirmed by IP assay using H460 lysates (Figures 5(g) and S3A). To further validate this reciprocal binding, lysates prepared from HEK293T cells transiently transfected with Flag-tagged TRIM6 and HA-tagged SLC1A5 were subjected to IP assay. Immunoprecipitation with anti-Flag or anti-HA antibodies brought down both Flag-TRIM6 and HA-SLC1A5, indicating that the two tagged proteins were associated with each other in HEK293T cells (Figures 5(h) and S3B). Taken together, we conclude that TRIM6 directly interacts with SLC1A5 to promote its degradation.

### 3.6. TRIM6 Regulates the Chemosensitivity of the Lung Cancer Cells In Vivo and In Vitro

**Figure 6:**
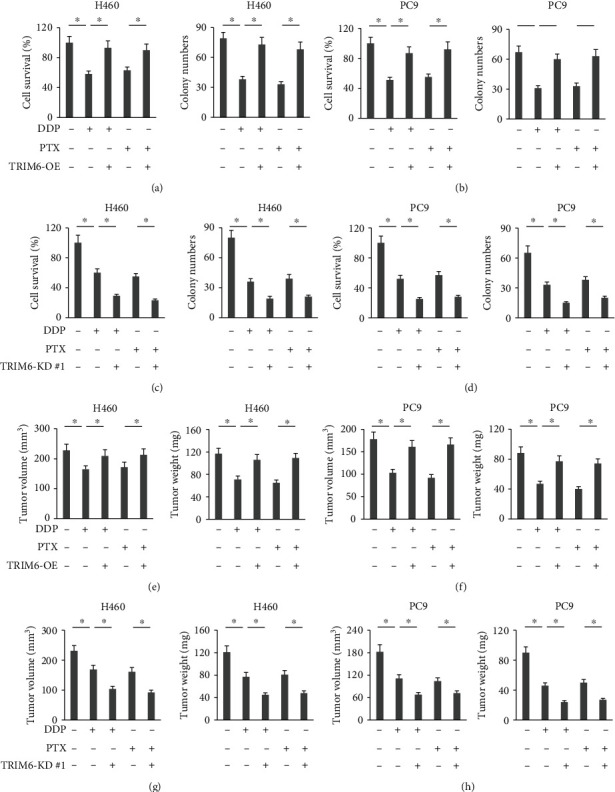
TRIM6 regulates the chemosensitivity of the lung cancer cells in vivo and in vitro. (a, b) Cell survival status and colony formation in DDP/PTX-treated lung cancer cells with or without TRIM6 overexpression (*n* = 6). (c, d) Cell survival status and colony formation in DDP/PTX-treated lung cancer cells with or without TRIM6 silence (*n* = 6). (e, f) Tumor volumes and weights in DDP/PTX-treated xenografts models inoculated with TRIM6-overexpressed lung cancer cells (*n* = 6). (g, h) Tumor volumes and weights in DDP/PTX-treated xenograft models inoculated with TRIM6-silenced lung cancer cells (*n* = 6). All data are reported as the mean ± SD, ^∗^*P* < 0.05 versus corresponding groups.

Given its effective role in regulating ferroptosis, we finally determined whether TRIM6 manipulation affected the chemosensitivity of the lung cancer cells in vivo and in vitro. As shown in Figures 6(a) and 6(b), TRIM6 overexpression significantly reduced DDP- and PTX-mediated toxic effects to H460 cells in vitro, as evidenced by the increased cell survival and colony formation. Conversely, TRIM6 silence potentiated the chemotherapeutic effects of DDP and PTX in H460 cells (Figures 6(c) and 6(d)). We also examined the role of TRIM6 on DDP- and PTX-mediated tumor-killing actions in mouse xenograft tumor models. In line with the in vitro findings, we observed that TRIM6 overexpression promoted, while TRIM6 knockdown further inhibited tumor growth upon DDP or PTX chemotherapy (Figures 6(e)–6(h)). To enhance the translational value of our findings, we also analyzed the predictive role of TRIM6 and SLC1A5 on patient survival in LUAD database. As shown in Figures S4A and S4B, both TRIM6 and SLC1A5 expressions negatively correlated with patient survival in LUAD database, indicating a clinical role of TRIM6 and SLC1A4 of lung cancer. These observations define TRIM6 as a promising therapeutic target for the treatment of lung cancer.

## 4. Discussion

The present study shows the role of TRIM6 on ferroptosis and chemosensitivity of lung cancer, and our major findings are presented as below. Firstly, TRIM6 is highly expressed in human lung cancer tissues and cells, and its expression in the lung cancer cells is further increased by ferroptotic stimulation. Secondly, TRIM6 overexpression inhibits, while TRIM6 silence promotes erastin- and RSL3-induced glutaminolysis and ferroptosis in the lung cancer cells. Thirdly, TRIM6 directly interacts with SLC1A5 to promote its ubiquitination and degradation, thereby inhibiting Gln import, glutaminolysis, lipid peroxidation, and ferroptotic cell death. Finally, TRIM6 overexpression reduces the chemotherapeutic effects of DDP and PTX. In contrast, TRIM6 silence sensitized human lung cancer cells to DDP and PTX in vivo and in vitro. Overall, our research for the first time defines TRIM6 as a negative regulator of ferroptosis in the lung cancer cells, and TRIM6 overexpression enhances the resistance of human lung cancer cells to chemotherapeutic drugs. Overall, TRIM6 is a promising therapeutic target for the treatment of lung cancer.

Ferroptosis, a newly identified form of cell death, plays critical roles in the development and chemoresistance of lung cancer. Wang et al. found that inhibiting ferroptosis facilitated the proliferation of human lung cancer cells, thereby promoting tumor progression [32], while inducing ferroptosis by erianin suppressed the growth and migration of the lung cancer cells [33]. Iron-related accumulation of lethal lipid ROS is the predominant feature during ferroptosis; however, we found that TRIM6 genetic manipulation did not affect Glu uptake, GSH synthesis, and iron transport. Gln is the most abundant amino acid in human tissues and plasma and provides nitrogen source for the biosynthesis of amino acids, nucleotides, and lipids. Besides, Gln is also an important carbon source and replenishes the intermediates for TCA cycle via glutaminolysis [14]. However, recent studies have reported that fueling of the TCA cycle by glutaminolysis accelerates lipid peroxidation and ferroptosis and that inhibiting glutaminolysis prevents ferroptotic cell death [15, 16]. SLC1A5, a membranous importer, is required for the uptake of neutral amino acids (e.g., Gln) and contributes to metabolic reprogramming of cancer cells [54]. Luo et al. proved that SLC1A5 suppression decreased Gln uptake, lipid peroxidation, and ferroptosis, thereby facilitating the survival of melanoma cells and tumor progression [15]. Consistently, we also found that TRIM6 directly interacted with SLC1A5 to promote its protein degradation and then inhibit erastin- or RSL3-mediated ferroptotic cell death. In contrast, TRIM6 silence elevated SLC1A5 expression and ferroptosis of the lung cancer cells.

TRIM family proteins function as kinds of E3 Ub-ligases and are implicated in the pathogenesis of lung cancer. Results from Chen et al. implied that TRIM28 reduced the proliferation of the lung cancer cell lines and that TRIM28 depletion led to increased cell proliferation [55]. Liu et al. found that TRIM29 knockdown suppressed the proliferation and invasion of human lung squamous cancer cells and also enhanced the chemosensitivity of DDP [56]. And knockdown of TRIM65 suppressed survival of DDP-resistant lung cancer cell lines and tumor growth [57]. TRIM6 belongs to the TRIM family and is well known for its role in the antiviral responses [24, 25]. Yet, recent studies revealed some additional actions of TRIM6, including the regulation on tumor progression [26–28]. Herein, we found that human lung cancer tissues and cells exhibited higher TRIM6 expression compared with the ANT or normal lung epithelial cell and that its expression in the lung cancer cells was further increased by ferroptotic stimulation. Consistently, Zheng et al. previously also detected an upregulated TRIM6 expression in human CRC samples [28]. Liu et al. determined that TRIM6 was highly expressed in angiotensin II- (Ang II-) stimulated fibrotic kidneys and positively correlated with the severity of renal fibrosis. Mechanistically, Ang II-induced ROS generation activated nuclear factor-*κ*B pathway, which subsequently elevated TRIM6 expression through binding to its promoter directly [58]. As we know, ROS overproduction is a key feature of myocardial ischemia/reperfusion (I/R) injury. Results from Zeng et al. revealed that cardiac TRIM6 mRNA and protein levels were significantly upregulated following I/R injury [26]. Based on these findings, we speculated that TRIM6 upregulated in ferroptotic condition might be associated with the increased oxidative stress. Meanwhile, we found that increased TRIM6 expression in the lung cancer cells upon ferroptotic stimulation could provide cytoprotective effects against chemotherapeutic reagents. Further detections revealed that TRIM6 reduced glutaminolysis via targeting SLC1A5-mediated Gln uptake. However, relatively little is known about how TRIM6 modulates SLC1A5 currently. TRIM6 acts as an E3 Ub-ligase and is essential for protein stability via regulating the ubiquitinated processes [24, 28]. In line with these studies, we proved that TRIM6 directly bound to SLC1A5 and promoted its ubiquinated modification at the posttranscriptional levels, thereby shortening the half-life of SLC1A5 protein and reducing ferroptotic cell death. Moreover, TRIM6 knockdown potentiated the lung cancer cells to DDP and PTX treatment in vivo and in vitro.

In summary, our findings determine a novel regulatory role of TRIM6 on ferroptosis and tumor progression of lung cancer. Genetic or pharmacological inhibition of TRIM6 may provide promising strategies for the treatment of lung cancer.

## Data Availability

The data that support the findings of this study are available from the corresponding author upon reasonable request.

## Supplementary Materials

Supplementary Materials Figure S1: TRIM6 knockdown does not affect the molecules essential for Glu uptake, GSH synthesis, and iron transport. Figure S2: SLC1A5 knockdown abrogates the protective effects against ferroptosis in TRIM6-deficient H460 cells upon erastin stimulation. Figure S3: TRIM6 directly interacts with SLC1A5. Figure S4: expressions of TRIM6 and SLC1A5 negatively correlate with patient survival in the LUAD database.
