# Supplementary material for: TRIM6 Reduces Ferroptosis and Chemosensitivity by Targeting SLC1A5 in Lung Cancer
Source: Oxid Med Cell Longev. 2023 Jan 9;2023:9808100. doi: 10.1155/2023/9808100 (PMC9842414; doi:10.1155/2023/9808100)
Supplement: Supplementary Materials — Figure S1: TRIM6 knockdown does not affect the molecules essential for Glu uptake, GSH synthesis, and iron transport. Figure S2: SLC1A5 knockdown abrogates the protective effects against ferroptosis in TRIM6-deficient H460 cells upon erastin stimulation. Figure S3: TRIM6 directly interacts with SLC1A5. Figure S4: expressions of TRIM6 and SLC1A5 negatively correlate with patient survival in the LUAD database. [file 9808100.f1.docx]

**TRIM6 reduces ferroptosis and chemosensitivity by targeting SLC1A5 in lung cancer**

Ying Zhang^1^ ^*^, Ping Dong^2^ ^*^, Nian Liu^3^, Jun-Yuan Yang^4^, Hui-Min Wang^5^, Qing Geng^2^

^1^ Department of Vascular Surgery, Renmin Hospital of Wuhan University, Wuhan, 430060, Hubei, China

^2^ Department of Thoracic Surgery, Renmin Hospital of Wuhan University, Wuhan, 430060, Hubei, China

^3^ Department of Neonatology, Renmin Hospital of Wuhan University, Wuhan, 430060, Hubei, China

^4^ Department of Gynecologic Oncology, Zhongnan Hospital of Wuhan University, Wuhan 430071, Hubei, China

^5^ Department of Fever Clinic, Renmin Hospital of Wuhan University, Wuhan 430060, Hubei, China

Correspondence: Qing Geng

Department of Thoracic Surgery,

Renmin Hospital of Wuhan University,

Jiefang Road 238, Wuhan, 430060, Hubei, China

Email: [gengqingwhu@whu.edu.cn](mailto:gengqingwhu@whu.edu.cn)


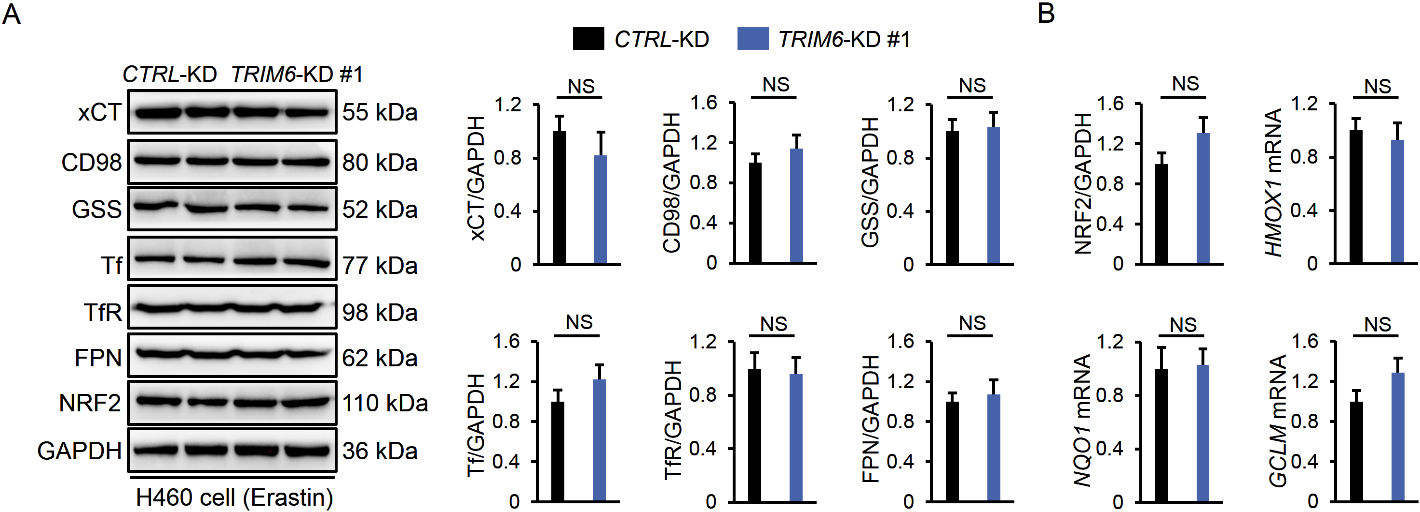


**Fig. S1. TRIM6 knockdown does not affect the molecules essential for Glu uptake, GSH synthesis and iron transport. (A)** Relative levels of proteins required for Glu uptake, GSH synthesis and iron transport (n=6). **(B)** NRF2 protein levels and the mRNA levels of its downstream targets (n=6). All data are reported as the mean ± SD. NS indicates no significance.


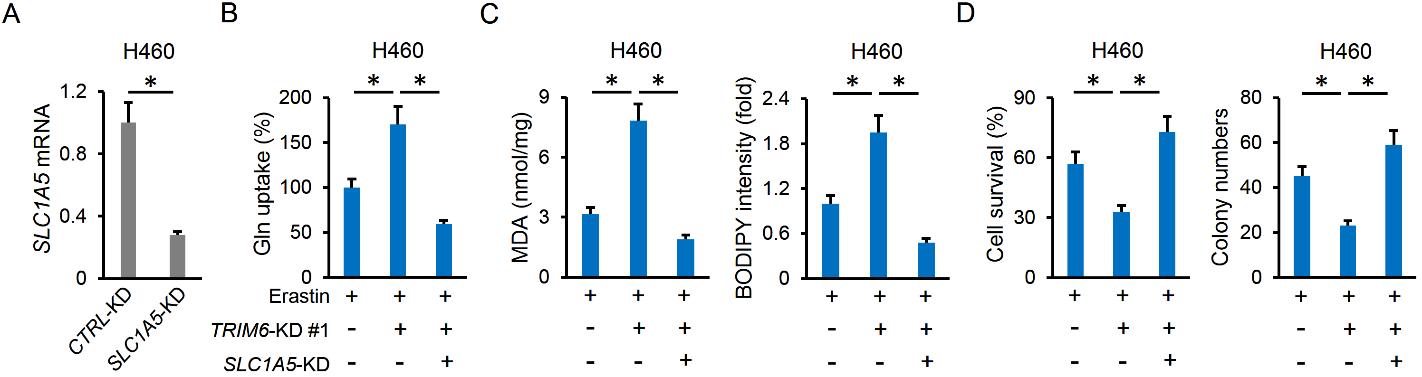


**Fig. S2. SLC1A5 knockdown abrogates the protective effects against ferroptosis in TRIM6-deficient H460 cells upon erastin stimulation. (A)** Relative *SLC1A5* mRNA levels in H460 cells with or without *SLC1A5*-KD infection (n=6). **(B)** Relative Gln uptake in erastin-treated H460 cells (n=8). **(C)** Intracellular lipid ROS levels and MDA formation in erastin-treated H460 cells (n=6). **(D)** Cell survival status and colony formation in erastin-treated H460 cells (n=6). All data are reported as the mean ± SD, **P* < 0.05 versus corresponding groups.


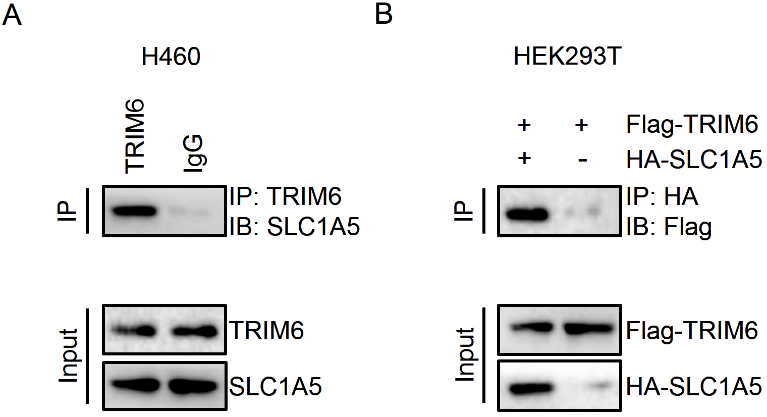


**Fig. S3. TRIM6 directly interacts with SLC1A5. (A-B)** IP assay for examining the interaction between TRIM6 and SLC1A5 (n=6).


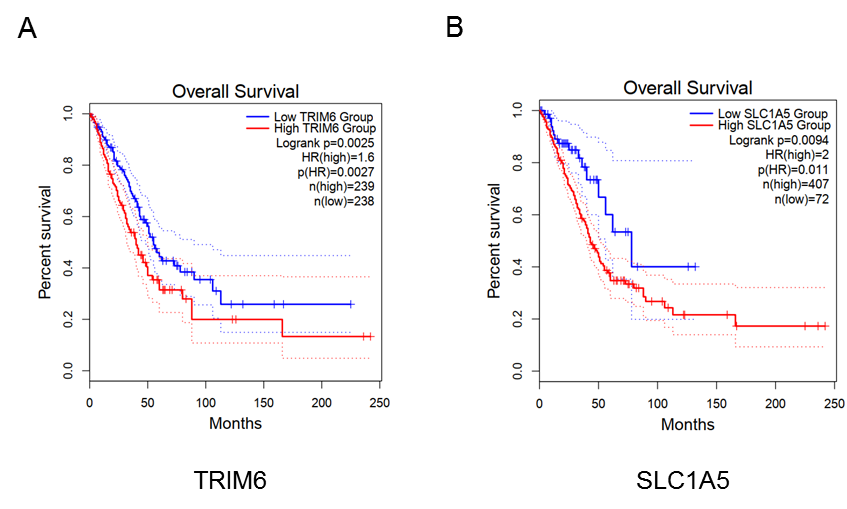


**Fig. S4. Expressions of TRIM6 and SLC1A5 negatively correlate with patient survival in LUAD database. (A)** Correlation between TRIM6 and patient survival (n=238-239). (B) Correlation between SLC1A5 and patient survival (n=72-407).
